# Can Creative Thinking Predict Academic Success in Medical Education? Correlating Torrance Test of Creative Thinking Scores and Five-year GPAs of Japanese Medical Students

**DOI:** 10.12688/mep.20889.1

**Published:** 2025-05-12

**Authors:** Marcellus Nealy, Hiroyuki Daida, Yuichi Tomiki, Dennis Dew, Takeo Higuchi

**Affiliations:** 1 Juntendo University, Chiba, Japan; 2 Juntendo University, Tokyo, Japan; 3 Mount Mercy University, Cedar Rapids, Iowa, USA; 4 Idea Marathon, Tokyo, Japan

**Keywords:** Torrance Test of Creative Thinking–Figural, creativity index, academic achievement, Japanese medical students, medical education, creative thinking

## Abstract

**Background:**

This study determined the correlation between creative thinking aptitude, measured by the Torrance Test of Creative Thinking–Figural (TTCT–F), and five-year academic achievement.

**Methods:**

The TTCT–F was administered to 135 first-year medical students at a Tokyo-based medical school in 2018. Participants’ academic records—annual GPAs over five years—were averaged, and data were analyzed in 2023. Pearson correlation coefficients examined the relationship between the TTCT–F Creativity Index and the five-year average GPA; multiple linear regression assessed the predictive value of TTCT–F components on GPA; canonical correlation analysis explored multivariate relationships.

**Results:**

The Creativity Index demonstrated a weak, non-significant correlation with the five-year average GPA. Fluency, Originality, and Elaboration components were not significantly correlated, while Abstractness of Titles demonstrated a moderate positive correlation. Linear regression indicated that Abstractness of Titles significantly predicted GPA, accounting for approximately 8% of the variance. Canonical correlation analysis revealed a moderate multivariate association between TTCT–F components and academic performance.

**Conclusions:**

Certain aspects of creative thinking, particularly abstract reasoning, may relate to academic success in medical education. Further research is needed to clarify the role of creative thinking in medical training and whether it warrants greater integration into curricula.

## Introduction

### Objective

This study seeks to determine whether there is a correlation between creative thinking aptitude, as measured by the Torrance Test of Creative Thinking – Figural (TTCT–F), and academic achievement in a Japanese medical school in Chiba and Tokyo prefectures over a five-year period, as measured by participants’ average five-year grade point average (GPA).

### Background

Healthcare is in constant flux. New diseases and complex health challenges require medical professionals to adapt quickly ( Dzau *et al.*, 2013; Hindin *et al.*, 2023). The COVID-19 pandemic was a powerful reminder of this truth. Innovative solutions were required to address many new problems: from developing diagnostic methods to establishing virtual care solutions ( Oyewole *et al.*, 2021; Patrucco *et al.*, 2021; Rando *et al.*, 2021; Woolliscroft, 2020). Indeed, the pandemic highlighted an urgent need for medical education to explore new techniques that nurture creative problem solving to prepare students for challenges requiring novel solutions ( Chen & Mullen, 2020).

### Creativity and medical education

Despite calls for further research on the role creative thinking plays in healthcare practice and education ( Ness, 2011; Patterson & Zibarras, 2017; Rodríguez *et al.*, 2019; Rohr *et al.*, 2021; Thabane *et al.*, 2023), studies remain scarce. Keyword searches of “creative thinking,” “divergent thinking,” “medical education,” “medical school,” “medical students,” and “medical curriculum” on PubMed, Google Scholar, and ProQuest reveal limited literature. While medical schools have begun to offer design thinking courses to foster creative thinking and problem solving ( McLaughlin *et al.*, 2019), to the best of our knowledge, the relationship between creative thinking and academic success in medical schools is seldom examined, particularly in Japan.

The literature offers little consensus on how to define creativity, a major roadblock for research on creative thinking. Kampylis and Valtanen (2010) identified 42 definitions and 120 collocations of creativity in their review of 1,090 journal articles and conference papers, 128 books, and 76 official documents and reports published between 1950 and 2009. These definitions ranged from “innovating something new” and “producing works of art” to “adeptly solving problems.” Of the 42 definitions presented, Torrance’s (1977, p. 6) definition aligns most closely with our conception: *“the process of sensing problems or gaps in information, forming ideas or hypotheses, testing and modifying these hypotheses, and communicating the results.”* This definition has practical value in the context of healthcare. For example, healthcare professionals gather information from patients and sense the problems or gaps in patients’ histories; a diagnosis or hypothesis is then proposed, tested, modified, and reported to patients or attending physicians for further adjustment. While Torrance’s definition of creativity may not encompass every experience within healthcare, it provides a solid starting point for this investigation. It also allows us to adopt a readily available tool for assessing creative aptitude: the Torrance Test of Creative Thinking (TTCT).

### Torrance Test of Creative Thinking

The TTCT is one of the most researched and widely applied instruments for gauging creative ability and has been translated into more than 35 languages ( Kim, 2011). Torrance’s longitudinal research has provided pivotal insights into the enduring influence of creativity. Torrance (2003) confirmed a strong correlation between TTCT scores and subsequent creative accomplishments. Later studies also demonstrate that the TTCT is a reliable indicator of creative aptitude ( Alabbasi *et al.*, 2022; Almeida *et al.*, 2008). Moreover, studies on its predictive validity reveal strong correlations between TTCT scores and academic performance. Kim and Lee (2020), reports a positive correlation between TTCT scores and academic success among engineering students in Korea, while Ayasrah *et al*. (2023) confirm the same for a sample of students at the Jordan University of Science and Technology. Kafipour *et al*. (2023) found that, of creativity, multiple intelligences, and motivation, creativity has the strongest correlation with academic achievement among Iranian students. Hence, creativity, as measured by the TTCT, has been shown to correlate with academic achievement across various educational contexts.

### Significance

The literature establishes a precedent linking TTCT with academic success in scientific fields, motivating our objective. Given the pivotal role of creative problem solving in medical innovation ( Adair, 2007; Munir & Awan, 2022), research on this relationship in the context of medical education should be expanded. We seek to address this gap through the present study.

## Methods

This section was written in accordance with the UK EQUATOR Centre’s EQUATOR STROBE checklist for cross-sectional studies ( von Elm *et al.*, 2007)

### Participants

We conducted a retrospective cohort study, administering the TTCT–F in 2018 as a proctored and timed test to a cohort of 135 first-year medical students at Juntendo University Faculty of Medicine in Chiba, Japan. The participants took the test simultaneously and were between the ages of 18 and 23 years old at the time. The cohort comprised 42 women (31.1%) and 93 men (68.9%) (see Table 1). The study was approved by the Juntendo University Institutional Review Board and conducted in accordance with ethical guidelines to ensure participant confidentiality and data anonymity. All participants provided informed consent before participation and allowed access to their academic records for research purposes. No exclusion criteria were applied, and all first-year medical students in the cohort were eligible to participate. Data were collected and stored in compliance with ethical guidelines to ensure confidentiality and anonymity.

**Table 1.** Demographic characteristics of participants (n = 135).

*Demographic * *variable*	*Category*	*Number of * *participants*	*Percentage * *(%)*
*Gender*	Female	42	31.11%
	Male	93	68.89%
*Age*	18	13	9.63%
	19	69	51.11%
	20	45	33.33%
	21	6	4.44%
	22	1	0.74%
	23	1	0.74%

### Instrumentation

We collected participants’ five-year average GPA, from 2018 to 2023. In cases where students were absent from the TTCT-F or where GPA records were incomplete, the missing data were handled using listwise deletion to maintain data integrity in statistical analyses. Our main tool was a Japanese translation of the TTCT–F, a standardized instrument designed to assess creativity through figural tasks. In 2011, Scholastic Testing Service Inc. (Bensenville, Illinois, USA) granted permission to a member of the present research team to translate and use the Torrance Tests of Creative Thinking – Figural (TTCT-F) for research purposes. Additionally, members of the research team obtained formal certification in TTCT scoring and interpretation from the Torrance Center for Creativity and Talent Development at the University of Georgia in 2012 and 2018.

The TTCT has two versions: a Verbal (TTCT–V) and a Figural (TTCT–F) test. The TTCT–F, which relies on images, is less biased for non-native English speakers, as the verbal test relies heavily on language and understanding of social and cultural context ( Kim, 2006). The TTCT–V is more difficult to translate while maintaining the original meaning, as translation is subject to interpretation. Since the TTCT–F only required translation of the instructions, this was less likely to affect the outcome. A comparison of the two tests further confirms the comprehensiveness and validity of the TTCT–F ( Kim, 2006; Kim, 2017; Torrance 1977). We thus chose it for assessing creative aptitude.

The TTCT–F has the following components:

- *Fluency* – assesses the ability to generate many ideas or solutions based on the number of drawings or ideas a person can create from a given stimulus.
- *Originality* – assesses the ability to produce unique and uncommon ideas, based on the novelty of responses compared with others.
- *Elaboration* – assesses the ability to add detail and further develop an idea by evaluating how well participants expand upon their initial ideas or drawings.
- *Abstractness of Titles* – assesses the ability to produce abstract and symbolic titles for drawings, reflecting deeper thinking and the ability to connect the drawing to broader or less obvious concepts.
- *Resistance to Premature Closure* – assesses the ability to keep an open mind and avoid hasty conclusions; by measuring how willing a person is to explore different possibilities rather than quickly concluding their drawings.

### Procedure

In 2018, 135 first-year Japanese medical students were gathered in one room under the supervision of faculty and administrators, including trained staff certified by the University of Georgia to administer the TTCT. A Japanese translation of the TTCT–F was provided by a third-party Japanese company specializing in TTCT administration and scoring. The test consisted of three sections: picture construction, picture completion, and lines. Each section was allotted 10 minutes, with 35–40 minutes allocated for the full test, including introduction, explanations, and wrap-up. After the TTCT–F, all related documents were collected, and scores were calculated by the company that provided the translation. The scores were then double-checked and verified by a TTCT-certified faculty member.

Over the next five years (2018–2023), after completion of the TTCT–F, participants’ academic records, specifically their annual GPAs, were collected from university records to calculate the five-year GPA. All data were anonymized to ensure participants’ confidentiality.

### Data analysis

Statistical analyses were performed using SPSS v. 30. The study size of 135 participants was determined based on the total available first-year medical student cohort, ensuring a comprehensive representation of the population. No formal power analysis was conducted, but the sample size was deemed sufficient for correlation and regression analyses. Pearson correlation coefficients were calculated to examine the linear relationship between the TTCT–F Creative Index scores and the scores for each of the TTCT–F’s five components listed in section 3.2 “Instruments.” To gauge the predictive potential of TTCT–F, multiple linear regression analysis was conducted to assess the extent to which Abstractness of Titles could predict the five-year GPA. Canonical correlation analysis was then performed to examine the multivariate relationships between the TTCT–F components and academic performance variables.

### Statistical significance

A significance level of p < 0.05 was adopted for all statistical tests. Standard diagnostic procedures were conducted to verify the validity of the regression models, including checks for: (a) **multicollinearity,** with variance inflation factors calculated to determine the degree of correlation among independent variables; (b) **homoscedasticity,** with residual plots examined to verify the assumption of equal variances; and (c) **normality of residuals,** with the normality of GPA scores assessed using the Kolmogorov–Smirnov and Shapiro–Wilk tests.

## Results

Table 2 presents the correlation between the overall TTCT–F Creativity Index and the five-year GPA average based on the Pearson correlation coefficient test. We observed a weak and statistically insignificant correlation (r = 0.067, p = 0.437), suggesting that, as a composite measure, the Creativity Index does not have a strong relationship with academic achievement in this sample.

**Table 2.** Pearson correlation coefficient: TTCT–F Creativity Index and five-year GPA Average.

*Correlations*		*GPA * *five-year * *average*
*TTCT–F Creativity Index*	*Pearson correlation*	0.067
	Sig. (2-tailed)	0.437
	N	135

*Note*. Correlation is significant at the 0.01 level (2-tailed); therefore, no significant correlation exists.

We then analyzed individual components of the TTCT–F to identify whether specific facets of creativity might correlate more strongly with GPA. Table 3 displays correlations for each TTCT–F component—Fluency, Originality, Elaboration, Abstractness of Titles, and Resistance to Premature Closure—with the five-year GPA average. Fluency, Originality, and Elaboration did not show statistically significant associations with GPA, while Abstractness of Titles demonstrated a moderate positive correlation (r = 0.284, p = 0.001). That is, students who scored higher in Abstractness of Titles, a measure reflecting the ability to see beyond the obvious, tended to have higher GPAs.

**Table 3.** Pearson correlation coefficient: Components of the TTCT–F Creativity Index and five-year GPA Average.

*TTCT–F components*	*Correlations*	*GPA five-year * *average*
*Fluency*	Pearson Correlation	-0.133
	Sig. (2-tailed)	0.125
	N	135
*Originality*	Pearson Correlation	-0.074
	Sig. (2-tailed)	0.396
	N	135
*Elaboration*	Pearson Correlation	0.061
	Sig. (2-tailed)	0.485
	N	135
*Abstractness of Titles*	Pearson Correlation	0.284
	Sig. (2-tailed)	0.001
	N	135
*Resistance to * *Premature Closure*	Pearson Correlation	-0.034
	Sig. (2-tailed)	0.698
	N	135
*GPA five-year average*	Pearson Correlation	1
	Sig. (2-tailed)	
	N	135

*Note*. Correlation is significant at the 0.01 level (2-tailed); therefore, no significant correlation exists in all components except Abstractness of Titles.

To further investigate the potential predictive power of Abstractness of Titles on GPA, a linear regression analysis was performed. Table 4 summarizes the regression model. Abstractness of Titles significantly predicted GPA, explaining approximately 8% of the variance (R² = 0.081, β = 0.284, p = 0.001). Thus, while marginal, this component has a statistically significant effect on academic achievement.

**Table 4.** Regression analysis model.

*Model*	*R*	*R square*	*Adjusted * *R square*	*Std. error of * *the estimate*	*Change*					*Durbin-Watson*
					*statistics*					
					*R square * *change*	*F change*	*df1*	*df2*	*Sig. F * *change *	
1	.284 ^a^	0.081	0.074	0.40107	0.081	11.647	1	133	0.001	1.911

*Note*. a. Predictors: (Constant), TTCT Abstractness of Titles; b. Dependent variable: GPA five-year average

Table 5 presents the results of the analysis of variance (ANOVA), which confirm the statistical significance of the regression model predicting GPA based on Abstractness of Titles (F = 11.647, p = 0.001). The ANOVA results strengthen the conclusion that abstract thinking, as a component of creativity, has a meaningful relationship with academic achievement.

**Table 5.** ANOVA for regression model significance.

*Model*		*Sum of squares*	*df*	*Mean square*	*F*	*Sig*
1	Regression	1.873	1	1.873	11.647	.001 ^b^
	Residual	21.394	133	0.161		
	Total	23.267	134			

*Note*. a. Dependent variable: GPA five-year average; b. Predictors (Constant), TTCT Abstractness of Titles

Table 6 shows the results of a canonical correlation analysis examining the multivariate association between the TTCT–F components and GPA. A moderate canonical correlation (r = 0.42, p = 0.015) with significant Wilks’ Lambda suggests a multivariate relationship between specific creative thinking components and academic performance, further supporting the importance of certain creative traits for academic achievement.

**Table 6.** Canonical correlation analysis of the components of TTCT–F and GPA.

*Canonical correlations*							
	*Correlation*	*Eigenvalue*	*Wilks statistic*	*F*	*Num D.F.*	*Denom D.F.*	*Sig.*

1	0.42	0.215	0.718	1.741	25	465.856	0.015
2	0.306	0.103	0.872	1.108	16	385.574	0.345
3	0.164	0.028	0.962	0.554	9	309.235	0.835
4	0.093	0.009	0.988	0.373	4	256	0.828
5	0.053	0.003	0.997	0.37	1	129	0.544

*Note*. The null hypothesis (H₀) for the Wilks test is that the correlations between the current and subsequent rows are 0.

Table 7 shows the results of the Kolmogorov–Smirnov and Shapiro–Wilk tests, which indicate a slight deviation from normality in the GPA distribution (p = 0.047). Nevertheless, the relatively large sample size (N = 135) enhances the reliability of the data. With a larger sample size, statistical estimates tend to be more robust to minor deviations from normality, reducing the likelihood non-normality would significantly affect the validity of correlation and regression analyses. Thus, the results can still be considered reliable in understanding the relationship between creative thinking components and GPA.

**Table 7.** Normality tests for GPA distribution.

*Kolmogorov–Smirnova*			*Shapiro–Wilk*		
*Statistic*	*df*	*Sig.*	*Statistic*	*df*	*Sig.*
0.075	135	0.047	0.98	135	0.047

*Note*. GPA five-year average: a Lilliefors significance correction

In conclusion, we found no significant correlation between the TTCT–F Creativity Index and academic achievement as measured by the five-year average GPA. However, Abstractness of Titles did have a moderate positive correlation with GPA, suggesting that it may be associated with higher academic performance. Further research is recommended to explore the underlying factors contributing to this association.

## Discussion

This study explored the relationship between creative thinking, as measured by the TTCT–F, and academic achievement, as represented by the five-year average GPA, in a cohort of Japanese medical students. Specifically, we examined the potential of the TTCT–F and its individual components, particularly Abstractness of Titles, to serve as predictors of academic success in medical education.

The overall TTCT–F Creativity Index does not have a strong or statistically significant correlation with GPA. However, the Abstractness of Titles component demonstrated a moderate positive correlation with GPA (r = 0.284, p = 0.001), suggesting that abstract thinking, as a cognitive skill, may play a unique and relevant role in academic success within the Japanese medical education context. It may potentially enhance students’ ability to synthesize information, make connections, and tackle problems in healthcare settings.

The study’s significance lies in its exploration of creativity within the context of Japanese medical education, providing original insights into the relationship between TTCT–F creative thinking metrics and academic success. By examining the role of TTCT–F in predicting academic success, this research opens a novel avenue for continued exploration on the benefits of integrating creative thinking into medical curricula and assessments. The mid- to long-term implications could lead to larger investigations that inform curriculum design in Japan and elsewhere, advocating for the integration of abstract and creative thinking into future medical education programs. Future research could also build upon these findings by evaluating other creative thinking assessment tools in this context. A better understanding of the relationship between creative thinking and academic achievement in medical school could also lead to valuable insights on ways to improve learners’ professional readiness.

### Importance of abstractness of titles

Abstractness of Titles, as measured by the TTCT–F, reflects a student’s ability to perceive relationships, connect ideas beyond the obvious, and generate meanings that go beyond surface-level interpretations ( Torrance, 2018). In medical education, these skills are crucial as they align closely with clinical competencies such as diagnostic reasoning, the ability to integrate complex information, and the capacity for flexible thinking in response to evolving patient presentations ( Mylopoulos *et al.*, 2018). Therefore, students who excel in abstract thinking may be better equipped to navigate the rigorous demands of medical training and practice.

In the context of Japanese medical education, where the curriculum is highly structured and traditionally focused on knowledge acquisition and technical proficiency ( Committee, 2022; Matsuyama *et al.*, 2016; Saiki *et al.*, 2017), the positive correlation between Abstractness of Titles and the five-year GPA average suggests that fostering creative thinking, particularly abstract reasoning, may enhance students’ educational outcomes.

### Insignificance of other TTCT–F components

Interestingly, other TTCT–F components, such as Fluency, Originality, Elaboration, and Resistance to Premature Closure, did not show statistically significant correlations with GPA. Fluency (the ability to generate a variety of ideas), Originality (the capacity to produce novel concepts), Elaboration (the skill of developing ideas in depth), and Resistance to Premature Closure (the tendency to avoid premature conclusions) may be undervalued in curricula that emphasize strict adherence to established protocols and medical guidelines. Japanese medical schools generally prioritize traditional medical knowledge and proven methods ( Onishi, 2018), which can limit opportunities for creative thinking and originality. However, the ability to elaborate ideas in detail is essential in medical practice, where physicians must diagnose, devise treatment plans, and communicate effectively with patients and their families. The tendency to draw premature conclusions may affect both academic success and medical practice. Therefore, both elaboration and resistance to premature closure would seem to have a role, even within a traditional medical curriculum. Despite these assumptions, our findings confirmed no statistically significant correlations between these components and the average five-year GPA. Without further research, the reasons for this remain speculative. Possibly, these skills, along with fluency and originality, are not fully assessed in the academic evaluations of Japanese medical schools. For example, multiple-choice or true-or-false exams may not be suitable for evaluating any of the TTCT–F categories, which could explain the lack of statistically significant correlations.

Note that Torrance (1962) also proposed the threshold theory, which posits a significant correlation between IQ and creativity, but only up to an IQ score of 120. This theory is not without critics ( Runco & Albert, 1986). Although IQ and GPA are distinct measures of intelligence, a similar threshold might exist in relation to academic achievement. However, this remains speculative. The reason why Abstractness of Titles showed a statistically significant correlation while other components did not can only be answered by further research within the context of Japanese and global medical education.

### Implications for medical education in Japan

The correlation between Abstractness of Titles and academic success suggests that fostering abstract thinking could benefit medical students by enhancing their academic and professional competencies. Incorporating opportunities for abstract reasoning and creative problem-solving may help students develop adaptability and integrative thinking. Assessing these skills could help educators gauge students’ progress in developing these essential abilities. This approach does not imply that creativity training should replace academic rigor but rather that it could complement existing learning methods to improve GPA and other academic outcomes, while better preparing students for real-world medical scenarios.

### Future directions

To explore the relationship between creative thinking and academic outcomes, as well as professional preparedness in medical education, incorporating both quantitative and qualitative measures could provide a more comprehensive understanding of how creativity supports medical training in Japanese medical schools. Further study of curriculum modifications that include assessments and exercises targeting abstract thinking may also reveal whether these skills translate into improved academic outcomes.

In conclusion, while the TTCT–F does not strongly correlate with academic success in medical education, specific components such as Abstractness of Titles may play a meaningful role. These results imply that fostering creative thinking, particularly abstract reasoning, could benefit medical students by enhancing skills that are crucial for both academic success and future clinical practice. Further investigation into curriculum adjustments aimed at nurturing abstract thinking may be valuable for improving learning outcomes in Japanese medical education.

### Limitations

This study has several limitations that should be acknowledged. First, the TTCT–F is just one measure of creativity and may only capture some dimensions of creative thinking relevant to Japanese medical education. Other types of tests, such as real-world problem-solving tasks, might provide a more comprehensive and context-appropriate view of creativity and its ability to predict academic success in medical school.

Second, as the TTCT–F measures creative potential rather than creative output, it may not have captured the type of creativity that translates directly into academic achievement in Japanese medical education. This could explain why TTCT–F scores did not show a strong correlation with GPA. Academic success may depend more on applied knowledge and technical proficiency than on potential for creative thinking. Although Alabbasi *et al*. (2022) support Kim’s (2017) suggestion to use only the TTCT–F if it was impractical to use both, they demonstrated a weak correlation between TTCT–V and TTCT–F, suggesting that both tests should be administered to fully assess students’ creative thinking ability.

Finally, the GPA primarily reflects performance on exams and coursework, which may undervalue qualities such as fluency, originality, elaboration, and resistance to premature closure. In addition to a wider range of creative thinking assessments, future research should also consider incorporating other measures of academic performance.

## Ethic and consent

This study, titled *"Torrance Test of Creative Thinking as a Predictor of Academic Achievement in Japanese Medical Students"*, was approved by the Juntendo University Institutional Review Board (Approval Number: E22-0431) on February 10, 2023 and conducted in accordance with the Declaration of Helsinki and Japan’s Ethical Guidelines for Medical and Health Research Involving Human Subjects.

Verbal informed consent was obtained from all participants. Verbal consent was approved by the ethics committee due to the following reasons: (1) the study posed minimal risk to participants, and (2) requiring written consent could potentially compromise anonymity, as the study design involved no collection of identifying information. Verbal consent therefore helped ensure participant confidentiality while reducing burden.

All participants granted permission to access their academic records for research purposes. Those who did not provide consent were given the option to opt out. Of the 140 eligible candidates, five chose not to participate, and their data were excluded from the study.

All data were collected and stored in compliance with ethical guidelines to ensure confidentiality and anonymity.

## Data Availability

Dryad: Can creative thinking predict academic success in medical education? Correlating Torrance Test of Creative Thinking scores and five-year GPAs of Japanese medical students. https://doi.org/10.5061/dryad.79cnp5j6p [ Nealy *et al*. (2025)] The project contains the following underlying data: 1. README.md 2. TTCT_GPA_Correlation_Raw_Data_2.csv Data are available under the terms of the Creative Commons Zero "No rights reserved" data waiver (CC0 1.0 Public domain dedication).?
