# Supplementary material for: Can Creative Thinking Predict Academic Success in Medical Education? Correlating Torrance Test of Creative Thinking Scores and Five-year GPAs of Japanese Medical Students
Source: MedEdPublish (2016). 2025 May 12;15:22. [Version 1] doi: 10.12688/mep.20889.1 (PMC12699210; doi:10.12688/mep.20889.1)
Supplement: Peer review history

# Reviewer report: Reviewer response for version 1

**DOI:** 10.21956/mep.22368.r44639

**Published:** 2025-12-11

**Authors:** Paul Garrud

**Affiliations:** 1 University of Nottingham, Nottingham, UK

- This study reports a predictive validity study that followed a cohort of medical students through medical school for five years and examined the relationship between the Torrance test of creativity, administered at the start of medical school, with their academic grades over the course of five years.
- There may be parallels (or, indeed, overlaps) between the Torrance concept of creative thinking and Croskerry’s dual process model of diagnostic reasoning (e.g. Croskerry , P. 2009 . Clinical cognition and diagnostic error: Applications of a dual process theory of reasoning . Advances in Health Sciences Education: Theory & Practice , 14 : 27 – 35 . 

- The study is described as a retrospective cohort, but it seems to be a prospective cohort study. 

- Choice of the Figural version of the Torrance test was well justified. 

- Ethical approval was obtained appropriately. 

- It can be useful to adjust significance levels when multiple comparisons are made a posteriori: however, the correlation observed between Abstractness of titles and 5-year mean GPA was moderate and significant at p<0.01 and, therefore, likely a real association. 

- The study does not, though, provide much basis for the suggested changes in Japan medical curricula. Rather, further research might look to see whether there is a valid association between diagnostic reasoning and the Abstractness of titles component of the Torrance test. Resistance to premature closure, for instance, might well be hypothesised as plausibly related to diagnostic errors. 

Have any limitations of the research been acknowledged?

Yes

Is the work clearly and accurately presented and does it cite the current literature?

Yes

If applicable, is the statistical analysis and its interpretation appropriate?

Yes

Are all the source data underlying the results available to ensure full reproducibility?

Yes

Is the study design appropriate and is the work technically sound?

Yes

Are the conclusions drawn adequately supported by the results?

Yes

Are sufficient details of methods and analysis provided to allow replication by others?

Yes

Reviewer Expertise:

Medical education, especially validity of selection tests.

I confirm that I have read this submission and believe that I have an appropriate level of expertise to confirm that it is of an acceptable scientific standard.

## References

[1] : Clinical cognition and diagnostic error: applications of a dual process model of reasoning. *Advances in Health Sciences Education* .2009;14(S1) : 10.1007/s10459-009-9182-2 27-35 10.1007/s10459-009-9182-2 19669918

# Reviewer report: Reviewer response for version 1

**DOI:** 10.21956/mep.22368.r43740

**Published:** 2025-10-01

**Authors:** Tina Kumra

**Affiliations:** 1 Johns Hopkins University School of Medicine, Baltimore, Maryland, USA

The study evaluates the correlation between student GPA averaged over five years and Torrance Test of Creative Thinking-Figural Index in a sample of 135 students at one medical school in Tokyo, Japan.  While the relationship overall between GPA and the Creativity Index was not found to be statistically significant, the investigators did find a statistically significant positive correlation between GPA and one component of the Index, Abstractness of Titles.  The manuscript is well written and organized with a thoughtful discussion that addresses limitations of the study.  I would suggest also including in the limitations the fact that the study population was conducted in one institution in Japan, with a sample size of medical students that may not be large enough to fully evaluate all possible correlations between GPA and the TTCT-F categories.

Have any limitations of the research been acknowledged?

Partly

Is the work clearly and accurately presented and does it cite the current literature?

Yes

If applicable, is the statistical analysis and its interpretation appropriate?

Yes

Are all the source data underlying the results available to ensure full reproducibility?

Yes

Is the study design appropriate and is the work technically sound?

Yes

Are the conclusions drawn adequately supported by the results?

Yes

Are sufficient details of methods and analysis provided to allow replication by others?

Yes

Reviewer Expertise:

Medical Education

I confirm that I have read this submission and believe that I have an appropriate level of expertise to confirm that it is of an acceptable scientific standard.
